# Targeted drug screening for autism based on Cav1.2 calcium ion channel

**DOI:** 10.1371/journal.pone.0324018

**Published:** 2025-05-29

**Authors:** Chao Liu

**Affiliations:** Dongguan City University, Dongguan, China; Abasyn University, Peshawar, Pakistan, PAKISTAN

## Abstract

This study presents a targeted virtual drug screening approach for autism spectrum disorder (ASD), focusing on Cav1.2 calcium ion channels as potential therapeutic targets. ASD is a complex neurodevelopmental disorder characterized by impairments in social communication and behavior, with genetic factors playing a significant role. Cav1.2 channels have been implicated in the pathophysiology of ASD due to their role in regulating neuronal excitability and synaptic transmission. We employed computational methods to virtually screen a large database of compounds for their potential to modulate Cav1.2 channel function. Molecular docking simulations were used to identify potential Cav1.2 inhibitors, followed by pharmacokinetic modeling to assess drug-like properties. Molecular dynamics (MD) simulations were performed to evaluate the interactions of the top candidates with Cav1.2, and Molecular Mechanics/Poisson-Boltzmann Surface Area (MM/PBSA) analysis was employed to predict binding free energies. This approach identified several promising drug candidates, including ZINC000828320609, which exhibited strong binding affinity to Cav1.2, favorable pharmacokinetic properties, and no predicted toxicity. The virtual screening results provide a solid foundation for further experimental validation and potential drug development for ASD, offering a novel and efficient strategy to target Cav1.2 channels in the treatment of this complex disorder.

## Introduction

Autism spectrum disorder (ASD) [1–3] is a complex neurodevelopmental condition that encompasses a broad range of social, communication, and behavioral deficits. Despite extensive research efforts, the pathogenesis of ASD remains poorly understood, and there is a lack of effective and targeted therapeutic options [4,5]. However, recent advances in genetics and neuroscience have begun to reveal potential molecular targets for drug development in ASD [6,7]. Among these, Cav1.2 calcium ion channels have emerged as promising candidates due to their critical role in neuronal excitability and synaptic transmission, as well as their involvement in ASD-related pathologies [8,9].

Cav1.2 channels, also known as L-type calcium channels, are voltage-gated calcium channels that regulate calcium influx into neurons [10–12]. They play a fundamental role in the control of neuronal excitability, neurotransmitter release, and gene expression [13,14]. Abnormalities in Cav1.2 channel function have been observed in ASD patients, suggesting that targeting these channels may provide a therapeutic avenue for treating ASD [15,16]. However, the complex nature of ASD and the lack of predictive animal models have hampered the drug discovery process [17–19].

Virtual drug screening represents a powerful tool in the drug discovery pipeline, especially for complex diseases like ASD. This approach utilizes computational methods to identify potential drug candidates from large databases of compounds, significantly reducing the time and cost of traditional experimental screening methods [20–22]. Virtual screening has been successfully applied to various diseases, including cancer, neurodegenerative disorders, and infectious disease [23–25]. However, its application to ASD has been limited due to the lack of well-validated targets and the heterogeneity of the disorder [26,27].

In this study, we aim to utilize targeted virtual drug screening to identify potential therapeutics for ASD based on Cav1.2 calcium ion channels. Our approach combines computational techniques such as molecular docking, pharmacokinetic modeling, MD and MM/PBSA to screen a large database of compounds for their potential to interact with Cav1.2 channels and modulate their function [28,29]. We hope to discover novel drug candidates that can restore normal Cav1.2 channel function and potentially provide therapeutic benefits in ASD.

The first step in our virtual screening process involves identifying potential Cav1.2 channel inhibitors from a large database of compounds. This is achieved through molecular docking simulations, which predict the binding affinity and interaction modes of compounds with the Cav1.2 channel’s active site. We utilize high-resolution structures of Cav1.2 channels obtained from AlphaFold prediction to ensure accurate prediction. Compounds that exhibit high binding affinity and favorable interaction modes are selected as potential drug candidates.

Next, we perform pharmacokinetic modeling to assess the drug-like properties of the selected compounds. This includes predicting their absorption, distribution, metabolism, excretion (ADME), and toxicity profiles [30,31]. Compounds that exhibit favorable pharmacokinetic properties [32,33] and low toxicity are prioritized for further analysis.

Finally, we utilize MD to evaluate the interactional effects of the selected compounds with the designated protein. After the simulation trajectory is analyzed, MM/PBSA is used to predict the binding free energies from MD simulation trajectory. Via these techniques we can gain insights into the potential mechanisms of action of the selected compounds and their potential therapeutic benefits in ASD.

**Fig 1:**
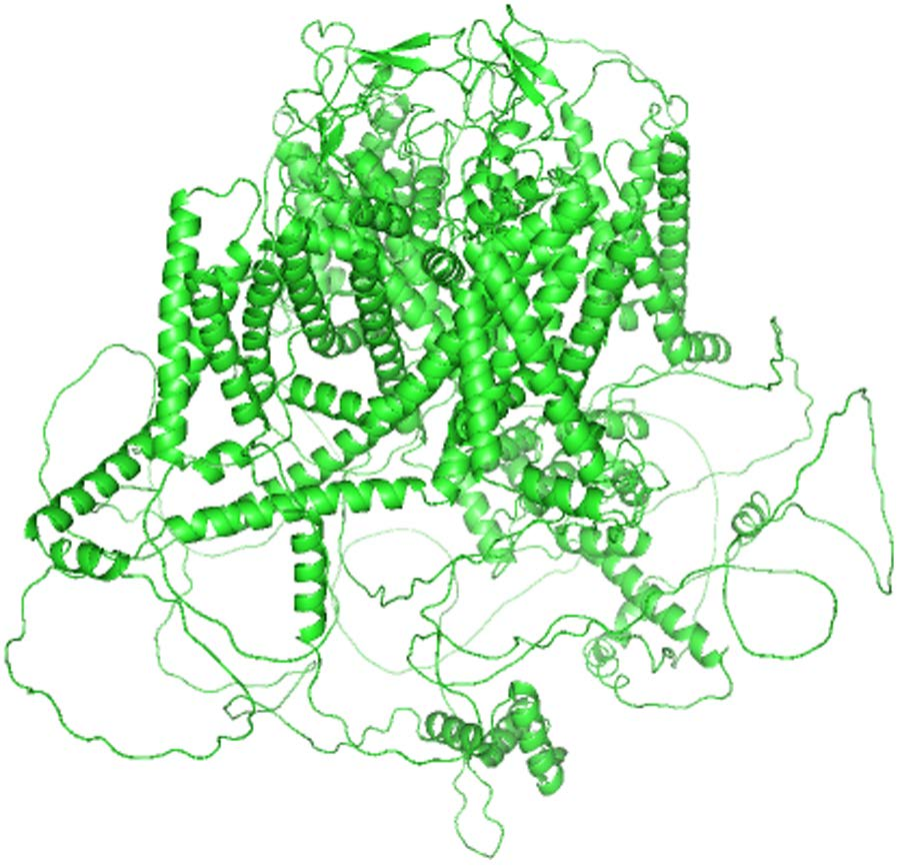
Schematic representation of the Cav1.2 calcium ion channel protein. The Cav1.2 calcium ion channel protein structure is acquired from Unirpot database predicted by AlphaFold.

In summary, the targeted virtual drug screening approach presented in this study aims to identify novel therapeutics for ASD based on Cav1.2 calcium ion channels. By combining computational techniques such as molecular docking, pharmacokinetic modeling, MD and MM/PBSA, we hope to discover drug candidates that can restore normal Cav1.2 channel function and provide therapeutic benefits in ASD. The successful application of this approach may pave the way for the development of effective and targeted therapeutics for this complex neurodevelopmental disorder. The specific structure of Cav1.2 calcium ion channel protein is shown in Fig 1.

## Materials and methods

### Dataset preparation

#### Drug library.

For our screening studies, we utilized the ZINC15 drug library. The ZINC (ZINC Is Not Commercial) database is a freely accessible collection of commercially available compounds that are useful for virtual screening and other drug discovery applications. The ZINC15 library specifically includes a diverse set of small molecules with known or predicted biological activities. Moreover, it has potential to cover a wide range of biological targets and activities.

#### Biological functions.

The compounds within the ZINC15 library are known to interact with a wide range of biological targets, including enzymes, receptors, ion channels, and other proteins involved in cellular signaling pathways. These interactions can lead to modulation of various biological processes such as cell proliferation, differentiation, apoptosis, inflammation, and neurotransmission, among others. The specific biological function of each compound in the context of our study was assessed based on its ability to inhibit Cav1.2 calcium ion channel protein.

By leveraging the ZINC15 library, we aimed to identify compounds with potential therapeutic relevance for ASD (Autism Spectrum Disorder). The library’s diversity and the known or predicted biological activities of its compounds make it a valuable resource for drug discovery and development.

#### Dataset acquisition.

Around 180,000 intricate molecules, each boasting a unique 3D framework, were procured from the vast ZINC15 repository. The selection criteria were stringent, encompassing the following points:

- **Polarity (logP) measurement**: Compounds with a suitable logP value (typically around 2–3) were chosen to balance aqueous solubility and lipid bilayer diffusion.
- **Charge**: Neutral compounds were selected to ensure compatibility with the docking simulations.
- **pH compatibility**: Compounds that were compatible with the physiological pH range were considered.
- **Commercial accessibility**: Only commercially available compounds were included to facilitate experimental validation.

Each of these molecules was a standard, readily available, and neutral lead-like drug candidates. After careful examination, the protonation status of these ligands was established, and crucial hydrogen atoms and charges were appended to ensure their readiness for further analysis.

### Molecular docking

The 3D coordinate of the Cav1.2 calcium ion channel protein structure was downloaded from the Uniprot protein data bank predicted by AlphaFold. The protonation state of the protein was determined and hydrogen atoms were added before molecular docking. The non-polar hydrogen atoms were then added into the connected heavy atoms to agree with the Autodock algorithm. The small molecules were docked into the target protein with the Autodock Vina v1.2.0 program. The Gasteiger partial charge was employed for both the macromolecules and small molecules because it could replicate about 80% of the crystallized binding poses of the co-crystallized ligands together with the scoring function. The grid spacing was hereby set to be centered in the active pocket, which is selected just exactly to cover the key amino acids. Each molecule was docked with 10 modes and the top docking pose with the lowest binding affinity was subjected to Molecular Mechanics/Poisson-Boltzmann Surface Area (MM/PBSA) rescoring calculations. For high performance computing, we employed MPI-Vina, a MPI based parallel implementation of Autodock Vina, for massive flexible docking tasks.

### ADMET prediction

ADMET properties play a significant role in determining the pharmacokinetic and pharmacodynamic profiles of compounds, and therefore, their potential as drug candidates. To predict ADMET properties, we utilized in silico tools and algorithms that have been developed and validated for this purpose. These tools typically employ machine learning or statistical methods trained on large datasets of known compounds with experimental ADMET data. Hereby SwissADME (www.swissadme.ch) server and ADMETLab (https://admetmesh.scbdd.com) were employed to evaluate ADME properties and toxicity of compounds from the docking results. SwissADME was chosen for its ability to predict a wide range of ADME properties, including logP, water solubility, and drug-likeness and ADMETLab 2.0 was employed for its comprehensive in silico toxicity testing, including drug-induced hERG toxicity, AMES toxicity, and carcinogenicity.

#### Pharmacokinetic (PK) parameters.

**LogPo/w (octanol-water partition coefficient):** LogPo/w predicts lipophilicity, influencing membrane permeability and oral bioavailability. Compounds with LogPo/w ~ 2–3 typically balance aqueous solubility and lipid bilayer diffusion.

**Molecular weight (MW):** MW impacts absorption (small MW favors diffusion) and renal clearance (large MW avoids filtration). MW < 500 Da is often preferred to enhance central neural system (CNS) penetration.

**Heavy atoms:** Heavy atoms refer to all atoms in a compound except hydrogen (H). These include carbon (C), nitrogen (N), oxygen (O), sulfur (S), and metal atoms. The number of heavy atoms directly influences a molecule’s lipophilicity (LogP) and synthetic accessibility.

**Arom. heavy atoms (aromatic heavy atoms):** Arom. heavy atoms denote heavy atoms embedded within aromatic rings (e.g., benzene, pyridine).These atoms contribute to molecular stability, binding interactions and drug-likeness.

**Rotatable bonds:** Rotatable bonds are single bonds (e.g., C-C, C-O) that allow free rotation between rigid molecular fragments (e.g., aromatic rings, aliphatic chains). They impact conformational flexibility, oral bioavailability and target selectivity.

**Hydrogen bond donors/acceptors:** H-bonds stabilize drug-target interactions. For Cav1.2 inhibitors, optimizing H-bond patterns improves potency and selectivity.

### Energy minimization

Energy minimization was employed to optimize the structural conformations of potential drug candidates and Cav1.2 receptor models. This step is crucial for ensuring accurate docking simulations and reliable binding energy predictions. After initial structure preparation, the compounds and receptor models were subjected to energy minimization procedures using Gromacs. This process involves iterative adjustments of the atomic positions and bond angles to achieve a state of minimal potential energy, representing a stable conformational state.

During energy minimization, constraints were applied to preserve critical features of the molecular structures, such as the integrity of chemical bonds and the geometry of functional groups. The minimization was performed using gradient-based algorithms that calculate the forces acting on each atom and iteratively adjust their positions to minimize the total potential energy. Usually steepest descent, conjugate gradients or L-BFGS (limited-memory Broyden-Fletcher-Goldfarb-Shanno) are involved.

By performing energy minimization, we were able to obtain optimized conformations of the drug candidates and Cav1.2 receptor models, which were then used in subsequent MD simulations. This approach helped to improve the accuracy and reliability of the screening process, enabling us to identify potential drug candidates with high affinity and specificity for the Cav1.2 calcium ion channels.

### Equilibration

After energy minimization, the prepared Cav1.2 calcium ion channel receptor models and potential drug candidates underwent equilibration procedures. This step began prior to simulating the molecular dynamics of the systems to achieve thermodynamic equilibrium.

Equilibration was conducted using molecular dynamics software Gromacs, where the systems were subjected to periodic boundary conditions and appropriate force fields. The simulations were run for sufficient time to allow the molecules to adapt to the solvent environment and relax into stable conformations.

During equilibration, various parameters such as temperature, pressure, and solvent composition were carefully controlled to mimic physiological conditions. By simulating the dynamics of the systems and allowing them to reach equilibrium, we were able to obtain stable conformations of the receptor-ligand complexes, which were further analyzed for potential drug-target interactions.

The first period is NVT equilibration which follows NVT ensemble, wherein 100-ps duration is employed. The second is NPT equilibration under NPT ensemble wherein 100-ps duration is also used.

### MD simulation

The MD simulation was initiated with coordinates acquired from AlphaFold prediction in the Uniprot database. The simulation was implemented with the software Gromacs 2019.6 package with the AMBER99SB-ILDN force field and TIP3P for protein and water respectively. The AMBER99SB-ILDN force field was selected for its accuracy in describing protein-ligand interactions and the TIP3P water model was used to simulate the aqueous environment surrounding the protein-ligand complex. A 100-nanosecond MD simulation was chosen to capture the essential dynamics of the protein-ligand interactions. Particle Mesh Ewald (PME) Method was implemented to handle long-range electrostatics with high accuracy.

The CHARMM-GUI web server was employed to add DPPC lipid bilayer membrane to the system and meanwhile proper number of sodium counter ions was added to neutralize this system. For molecular visualization VMD and XMGRACE were utilized.

RMSD (Root Mean Square Deviation) is a widely used metric in molecular dynamics simulations to quantitatively assess the deviation of a molecular structure from a reference structure. It measures the average distance between corresponding atoms in two structures, typically the initial structure and the structure at a given time point during the simulation. RMSD calculations are crucial in analyzing the stability and dynamics of biomolecules such as proteins and nucleic acids. A low RMSD value indicates that the molecular structure remains close to the reference, while a high RMSD value suggests significant structural changes have occurred. By plotting RMSD as a function of time, researchers can track the evolution of a molecular system and identify potential conformational transitions or regions of stability. RMSD is a valuable tool in structural biology, biochemistry, and drug discovery, enabling insights into the behavior of biomolecules at the atomic level.


$$RMSD=\sqrt{\frac{\sum_{i=0}^{N}\left[m_{i}{*\left(X_{i}-Y_{i}\right)}^{2}\right]}{M}}$$

RMSF (Root Mean Square Fluctuation), is a measure of the magnitude of atomic fluctuations within a molecular structure over a period of time, typically obtained from molecular dynamics simulations. It quantifies the average deviation of each atom from its mean position and provides an estimate of the dynamic flexibility and stability of different regions within the structure. RMSF is calculated by taking the square root of the mean of the squared differences between the instantaneous positions of an atom and its average position over the simulation time. By plotting RMSF values as a function of the atom’s position in the molecule, researchers can visualize regions that exhibit high or low mobility, such as flexible loops or rigid domains. RMSF is a valuable tool in the analysis of biomolecular dynamics, helping scientists understand the functional roles of different regions within proteins, nucleic acids, and other biomolecules. It can provide insights into mechanisms of protein function, allosteric effects, and drug-target interactions.


$$RMSF=\sqrt{\frac{1}{T}\sum_{t=1}^{T}\sum_{i=1}^{N}{\left(x_{i}(t)-\overset{―}{x_{i}}\right)}^{2}}$$

The moment of inertia, also known as the rotational inertia or angular mass, is a measure of a body’s resistance to changes in its rotational motion about a given axis. It quantifies the distribution of mass with respect to an axis of rotation, with a higher moment of inertia indicating a greater resistance to rotational acceleration. The moment of inertia plays a crucial role in various physical phenomena, including rotational dynamics, torque, and angular momentum. It is represented by the radius of gyration, denoted by *k*, formulated as follows:


$$k=\sqrt{\frac{I}{m}}$$

#### Molecular dynamics (MD) simulation parameters.

**Temperature:** Temperature controls molecular motion and interactions during MD simulations. By mimicking physiological conditions, we assess the stability of drug-Cav1.2 complexes under biologically relevant thermal fluctuations, critical for predicting in vivo binding stability.

**Force field:** Force fields mathematically describe atomic interactions (bonded/nonbonded). The choice of force field directly impacts the accuracy of protein-ligand complex geometry and dynamics, ensuring reliable free energy calculations.

**Solvent model:** Explicit solvent models (TIP3P) or implicit solvation (Poisson-Boltzmann) account for water-mediated effects on binding. Solvent influences ligand desolvation penalties and entropy changes, critical for binding affinity predictions.

### Per-residue energy decomposition using MM/PBSA

The MM/PBSA method was used to decompose the binding free energy into various components, including electrostatic energy, van der Waals energy, and nonpolar solvation energy. This allowed for a detailed understanding of the forces driving the protein-ligand interaction.

Per-residue energy decomposition using MM/PBSA is a computational method that provides a detailed breakdown of the energetic contributions to the binding affinity between two molecules, such as a protein and a ligand, at the level of individual residues. MM/PBSA, which stands for Molecular Mechanics/Poisson-Boltzmann Surface Area, combines molecular mechanics (MM) calculations of the internal energies of the molecules with continuum solvent models to estimate the solvation free energy.

In per-residue energy decomposition, the total binding free energy is partitioned into contributions from each residue in the protein. This allows researchers to identify key residues that contribute significantly to the binding affinity, providing insights into the mechanisms of molecular recognition and binding specificity. The per-residue energy decomposition can be used to guide drug design and optimization efforts, aiming to enhance the affinity and selectivity of small molecule ligands for their target proteins.

The free energy of binding (Δ*G*_binding_) represents the change in free energy that occurs when two molecules, such as a ligand and a receptor, bind to each other, forming a stable complex.


$$\Delta G_{binding}=\Delta H-T\Delta S$$

The values for Δ*H* and Δ*S* are obtained through a set of methods. Hereby Δ*G*_binding_ follows the relationship:


$$\Delta G_{binding}=\;\left(\Delta E_{MM}+\;\Delta G_{PB}+\;\Delta G_{non-polar}\right)-T\Delta S$$

where Δ*E*_MM_, Δ*G*_PB_ and Δ*G*_non-polar_ are the molecular mechanics energy, the Poisson-Boltzmann energy and the non-polar solvation energy, respectively. The latter two terms are usually referred to as Δ*G*_PBSA_. Δ*E*_MM_ comprises terms for internal, van der Waals and coulombic energies which are computed via Molecular Mechanics force field. The ligand, receptor and complex are exposed to MM/PBSA analysis in explicit solvent MD simulations.

MM/PBSA calculation is a computational method that combines molecular mechanics (MM) calculations of the internal energies of molecules with continuum solvent models, such as the Poisson-Boltzmann equation and surface area term, to estimate the solvation free energy and thereby predict the binding affinity between two molecules.


$$\Delta H=\Delta E_{MM}+\;\Delta G_{PB}+\;\Delta G_{non-polar}$$

The non-polar solvation free energy was in linear relationship with the solvent-accessible surface area (SASA):


$$\Delta G_{non-polar}=\gamma SASA+\beta$$

This whole analysis was performed to identify key residues involved in ligand binding, providing insights into the mechanisms of molecular recognition and binding specificity.

#### Binding energy calculation parameters (MM/PBSA).

**Enthalpy(ΔH):** Enthalpy reflects the total energy change (heat transfer) during binding. Negative ΔH indicates favorable exothermic interactions (e.g., hydrogen bonds, hydrophobic contacts) while positive value infers endothermic interactions.

**Entropy (ΔS):** Entropy quantifies disorder changes. Ligand binding often restricts conformational freedom, leading to negative entropy contributions (ΔS < 0), which oppose binding despite favorable enthalpy.

**Free energy of binding (ΔG):** ΔG = ΔH – TΔS. A negative ΔG indicates spontaneous binding, with magnitude correlating to affinity. MM/PBSA decomposes ΔG into polar (electrostatic) and nonpolar (hydrophobic) components, aiding rational drug optimization.

## Results and discussion

### Virtual screening

Amid the vast ZINC15 database, we navigated to approximately 180,000 potential drug candidates. Utilizing cutting-edge virtual screening techniques, we identified the most promising molecule to target the receptor among these, the cream of the crop emerged — 10 compounds, boasting binding affinities ranging from −9.7 to −10.2 kcal/mol. In Table 1, we showcase the top 10 compounds. We now embark on a deeper analysis, scrutinizing their absorption, distribution, metabolism, excretion (ADME) properties, and toxicity to refine our selection.

**Table 1:** Docking results of MPI-Vina between protein and ligands.

ZINC ID	Short name in this study	Binding Affinity(kcal/mol)
**ZINC000013923892**	**Lig1**	**−10.2**
**ZINC000252641347**	**Lig2**	**−9.9**
**ZINC000004697718**	**Lig3**	**−9.9**
**ZINC000002685589**	**Lig4**	**−9.8**
**ZINC000186886036**	**Lig5**	**−9.8**
**ZINC000003057249**	**Lig6**	**−9.8**
**ZINC000013759087**	**Lig7**	**−9.7**
**ZINC000000082103**	**Lig8**	**−9.7**
**ZINC000828320609**	**Lig9**	**−9.7**
**ZINC000000268095**	**Lig10**	**−9.7**

### ADMET prediction

In our study, we harnessed the power of the SwissADME server to explore the crucial ADME properties of our compounds. Specifically, we delved into their lipophilicity — their ability to dissolve in fats, oils, and nonpolar solvents — as well as their water solubility and overall drug-likeness. These metrics, captured in Table 2, provide invaluable insights into the potential druggability of our top 10 ligands (denoted as Lig1 to Lig10 in accordance with Table 1). Our findings indicate that all these compounds exhibit promising properties for drug development.

**Table 2:** List of ADME properties of top 10 ligands.

Properties		Lig1	Lig2	Lig3	Lig4	Lig5	Lig6	Lig7	Lig8	Lig9	Lig10
Physicochemical properties	MW(g/mol)	329.32	323.31	323.31	304.31	345.39	346.38	317.34	320.3	348.35	336.41
	Heavy atoms	25	24	24	23	26	26	24	24	26	24
	Arom. heavy atoms	19	17	17	19	12	10	15	16	12	16
	Rotatable bonds	2	5	5	3	3	2	2	2	2	4
	H-bond acceptors	5	6	6	5	3	4	3	5	4	3
	H-bond donors	2	1	1	2	2	1	2	1	2	1
Lipophilicity	Log Po/w	1.51	2.01	2.01	1.91	2.21	2.74	2.35	1.97	2.53	2.43
Water solubility	Log S (ESOL)	Soluble	Moderately soluble	Moderately soluble	Soluble	Soluble	Moderately soluble	Moderately soluble	Moderately soluble	Moderately soluble	Moderately soluble
Pharmacokinetics	GI absorption	High	High	High	High	High	High	High	High	High	High
Drug-likeness	Lipinski	Yes	Yes	Yes	Yes	Yes	Yes	Yes	Yes	Yes	Yes
Medi. Chemistry	Synth. Accessibility	3	3.06	3.06	3.05	2.99	4.05	2.98	3.41	3.62	2.83

However, drug discovery is a multifaceted journey, and we needed to ensure that our compounds did not harbor any undesirable side effects. To this end, we turned to ADMETLab 2.0, a web-based tool that specializes in in-silico toxicity testing. Here, we examined our top 10 compounds for drug-induced hERG toxicity, AMES toxicity, and carcinogenicity. As shown in Table 3, only ZINC000004305360 (Lig9) emerged unscathed, passing all the toxicity tests. This compound, therefore, became the focus of our subsequent analysis, promising to be a safe and effective drug candidate.

**Table 3:** List of the drug-induced hERG inhibition, AMES toxicity, carcinogens of top 10 ligands.

ZINC ID	hERG inhibition	AMES	Carcinogens
ZINC000013923892	No	No	Yes
ZINC000252641347	No	Yes	Yes
ZINC000004697718	No	Yes	Yes
ZINC000002685589	No	Yes	Yes
ZINC000186886036	No	No	Yes
ZINC000003057249	No	Yes	Yes
ZINC000013759087	No	Yes	No
ZINC000000082103	No	No	Yes
ZINC000828320609	No	No	No
ZINC000000268095	No	No	Yes

### Energy minimization and equilibration

**Fig 2:**
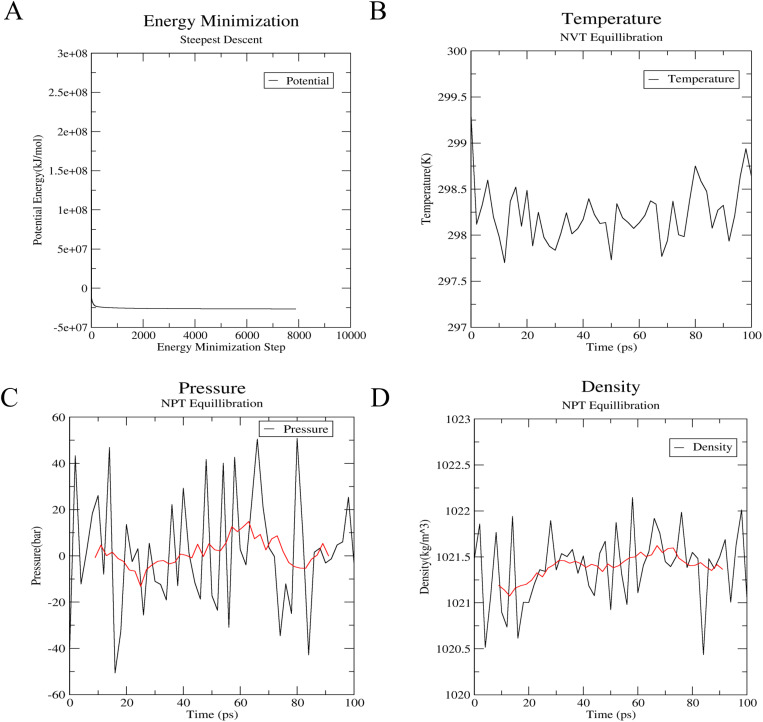
The energy minimization and equilibration step before MD simulation. (A) The variation of potential energy with energy minimization step. (B) The variation of temperature within 100 ps. (C) The variation of pressure within 100 ps. (D) The variation of density within 100 ps.

The simulation setup was carefully crafted, utilizing the AMBER99SB-ILDN force field for protein and the TIP3P model for water. A cubic water box surrounded and solvated the protein-water complex, initiating a series of refinement steps. Initially, a minimization process with about 8,000 steps ensured that the system energy was optimized, using a steep descent integrator and a fine 0.001 ps time step. As depicted in Fig 2A, the potential energy underwent a rapid descent, starting at 3.00 × 10^8^ kJ/mol and stabilizing at −4.50 × 10^7^ kJ/mol after approximately 80 steps, indicating a well-converged system.

Following energy minimization, the system underwent a gradual heating process, depicted in Fig 2B. Utilizing the NVT ensemble, the temperature was smoothly increased to 300K over 100 ps, with initial velocities assigned according to a Maxwellian distribution. The temperature initially fluctuated significantly but eventually stabilized around 300K, demonstrating thermal equilibration.

Moving to the next phase of equilibration, the system underwent pressure equilibration in the NPT ensemble, as shown in Fig 2C. The Parrinello-Rahman barostat effectively maintained pressure coupling, resulting in a mean pressure of 1.0 ± 30.1 bar, with the reference set at 1 bar. The large root-mean-square fluctuation in pressure is typical during molecular dynamics simulations, making the small difference between the obtained average and the reference value statistically insignificant. The 10-ps running average highlights the trend of pressure variation over time.

Concurrently, the density of the system was monitored, as shown in Fig 2D. The experimental density of water is 1000 kg/m3, while the theoretical density of the SPC/E water model is 1008 kg/m3. Our simulated average density over the entire simulation was 1021 ± 1 kg/m3, closely aligning with both experimental and theoretical values. This close agreement validates the accuracy and success of the simulation process.

After the system has achieved thermal and pressure equilibrium, the position restraints are poised to be liberated, heralding the commencement of the momentous molecular dynamics (MD) simulation. Prepared for data collection, the MD simulation will span an impressive 100 nanoseconds, a testament to the rigor and precision of our computational methods.

To accurately handle the long-range electrostatics within the system, we have implemented the Particle Mesh Ewald (PME) method, setting the PME order to 4. This ensures that electrostatic interactions are computed with utmost accuracy, capturing even the finest details of the system’s dynamics.

As the simulation progresses, the program will exercise its intelligence to determine the optimal number of processors required for the PME computations. This ensures that the computational load is evenly distributed, maximizing efficiency and minimizing resource utilization.

To further accelerate the computations, we have harnessed the power of GPU accelerators on a state-of-the-art supercomputer. These high-performance processors work in tandem with our PME algorithm, enabling us to traverse the vast simulation timeline with unprecedented speed and accuracy.

In summary, the release of position restraints, the execution of a 100-nanosecond MD simulation, the implementation of PME with optimized processor allocation, and the utilization of GPU accelerators all contribute to a robust and efficient computational pipeline, ensuring that we capture the essence of the system’s dynamics with unparalleled precision.

### Unrestrained MD simulations

**Fig 3:**
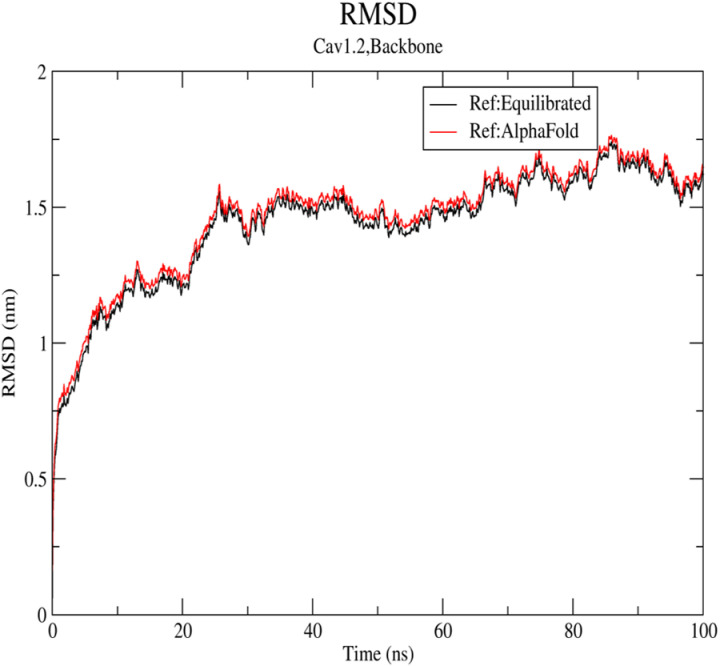
The RMSD values extracted from the complex structure within 100 ns MD simulation time.

RMSD measures the deviation of the protein structure from its initial conformation over the course of the simulation. The RMSD values for Lig9 exhibited a notably lower deviation compared to other candidates, indicating that Lig9 maintains a stable structure when bound to Cav1.2. This stability is crucial for effective inhibition of the channel. Most importantly, Fig 3 demonstrates Lig9 possesses slightly stable structure compared to the one present in the minimized, equilibrated system due to its slight lower RMSD.

**Fig 4:**
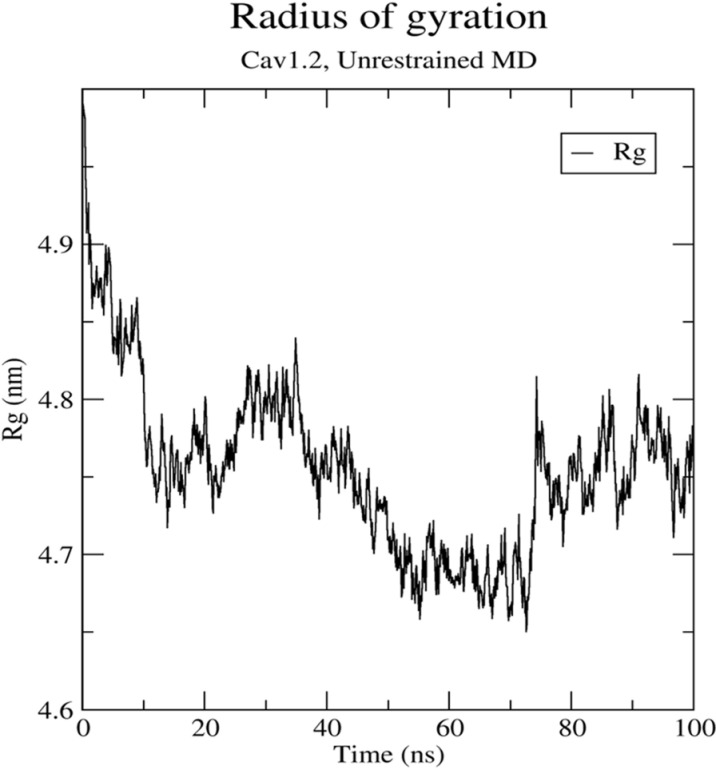
The radius of gyration values extracted from the complex structure within 100 ns MD simulation time.

Additionally, our analysis of the radius of gyration of Cav1.2 revealed a gradual decrease from 4.97 nm to 4.75 nm with a certain oscillation during the simulation period, as shown in Fig 4. The radius of gyration measures the compactness of the protein structure. During the simulation, the radius of gyration of Cav1.2 showed a gradual decrease with some oscillation, indicating that the protein structure became more compact when Lig9 was bound. This compactness suggests a more stable protein-ligand complex.

**Fig 5:**
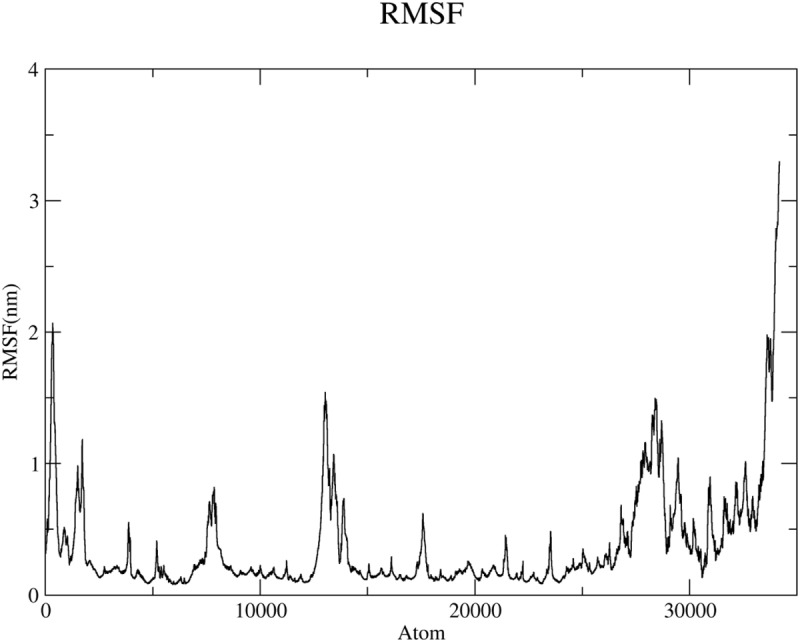
The RMSF values extracted from the complex structure within 100 ns MD simulation time.

RMSF measures the average atomic fluctuations within the protein structure, providing information about its flexibility. To further delve into the dynamics of the Cav1.2 protein, we calculated the root mean square fluctuation (RMSF) of its constituent atoms based on the simulated trajectories. As depicted in Fig 5, Lig9 binding results in significantly reduced RMSF values for most atoms, particularly those with the three highest fluctuations. This indicates that Lig9 promotes a more rigid and stable conformation of Cav1.2, potentially enhancing its inhibitory effects.

### MM/PBSA free energy calculation

MM/PBSA analysis was employed to predict the binding free energy (ΔG_binding_) of the protein-ligand complex and to decompose this energy into individual energetic components. Utilizing the MM/PBSA method, we were able to assess the free energies involved in the interactions between our protein models. This approach, implemented through the gmx_MMPBSA program, offers a semi-quantitative lens into the stability of these systems (Poli et al., 2020). As indicated in Table 4, various energy components contribute to the overall binding free energy, with the van der Waals energy (VDWAALS) standing out as the primary favorable contributor. The electrostatic energy (EEL) also plays a significant role, while the nonpolar solvation energy (ENPOLAR) contributes slightly but favorably. Specifically, the 1–4 interactions of the electrostatic and van der Waals energies further emphasize the importance of these forces. The Poisson-Boltzmann energy (EPB) and dispersion energy (EDISPER) also factor into the overall picture.The gas phase Gibbs free energy G_gas_ contributes favorably to the total energy while the liquid phase G_solv_ behaves opposite.

**Table 4:** List of energy components(kcal/mol).

Energy component	Average	σ	SEM
BOND	0.00	0.00	0.00
ANGLE	0.00	0.00	0.00
DIHED	0.00	0.00	0.00
VDWAALS	−41.00	3.18	0.77
EEL	−38.12	7.91	1.92
1-4 VDW	0.00	0.00	0.00
1-4 EEL	0.00	0.00	0.00
EPB	56.00	3.96	0.96
ENPOLAR	−4.10	0.10	0.02
EDISPER	0.00	0.00	0.00
ΔG_gas_	−79.13	8.06	1.95
ΔG_solv_	51.90	4.01	0.97
ΔG_total_	−27.23	5.48	1.33

**Fig 6:**
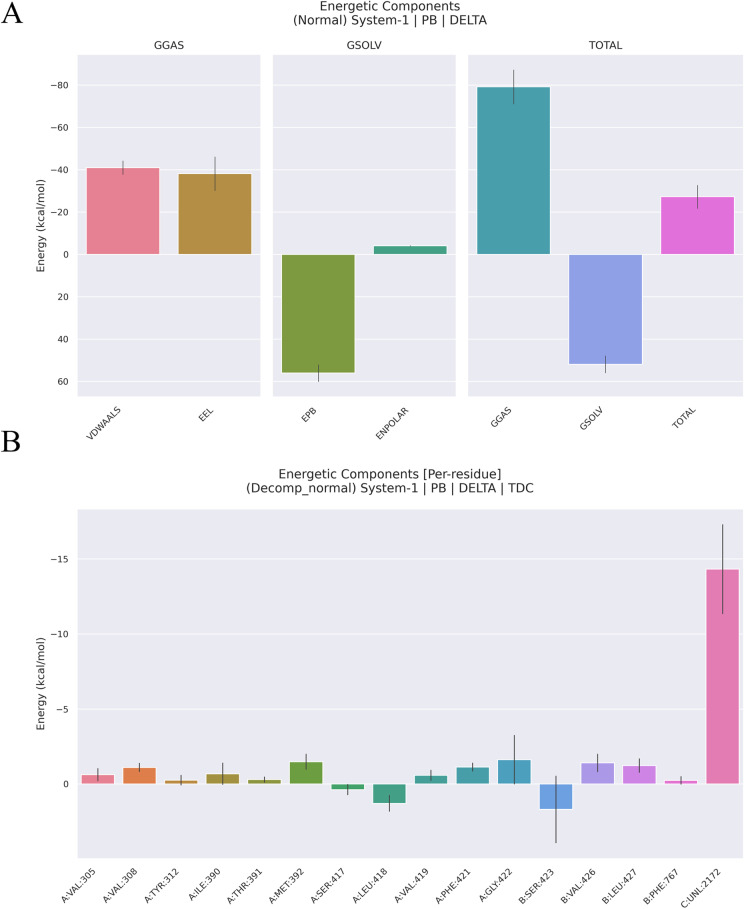
The energy decomposition details. (A) The illustration of energy difference for the receptor-ligand complex. (B) Per-residue decomposition energies for the Cav1.2 protein using MM/PBSA. VDWAALS denotes the van der Waals energy, EEL denotes the electrostatic energy, ENPOLAR denotes the nonpolar solvation energy and EPB denotes the Poisson-Boltzmann energy.

The preeminence of electrostatic energy in our results underscores the critical role it plays in stabilizing the complex between Cav1.2 and Lig9. This electrostatic force is pivotal in favoring the formation and maintenance of the homo-hexameric structure. Visualizing these energies, as presented in Fig 6A, provides a compelling confirmation of our findings.

To gain deeper insights into the intricacies of these protein-ligand interactions, we further performed MM/PBSA decomposition analyses. As Fig 6A demonstrates, the relative gas phase Gibbs free energy (ΔGgas) emerges as the dominant component, while the nonpolar solvation energy (ENPOLAR) occupies the least significant position. These insights not only validate our previous conclusions but also provide a more nuanced understanding of the forces that govern these vital interactions. In summary, our results convincingly demonstrate that Lig9 can effectively bind to the protein Cav1.2, highlighting the importance of electrostatic and van der Waals forces in this process.

In Fig 6B, we delve deeper into the intricate energetic landscape of the Cav1.2 protein, courtesy of the MM/PBSA per-residue decomposition. This analysis reveals that certain residues stand out as the true guardians of the protein’s function, namely GLY422, VAL426, and LEU427. These titans contribute significantly to the overall energetic stability of the protein, their importance underscored by their conservation across the Cav1.2 protein.

Notably, these residues are not just passive participants; they actively engage in ligand binding. This suggests that these residues form a key interface for the protein to interact with and regulate its environment.

## Conclusion

In this meticulous study, researchers harnessed the power of bioinformatics to identify potential regulators of the Cav1.2 protein. Cav1.2 stands as a gatekeeper of cations, facilitating their exit from cells in times of distress, such as cellular swelling. This is pivotal in maintaining cellular equilibrium against osmotic fluctuations. This equilibrium is essential for cells to function optimally. The protein structure is obtained from AlphaFold prediction with an accuracy of over 80% rather than an experimental result. The benefit comes from its possibility for drug screening without experimental structure. However, there lies in potential limitations and uncertainties in this study due to this not-fully-accurate predicted structure.

Using virtual screening, the researchers sifted through a vast library of over 180,000 compounds, seeking those with the highest affinity for Cav1.2. After rigorous evaluation, ZINC000828320609 emerged as the champion, surpassing all others based on toxicity predictions. Its binding pose and interactions with the protein were thoroughly scrutinized.

To gain deeper understanding of how ZINC000828320609 interacts with Cav1.2, researchers employed molecular dynamics (MD) simulations. The simulations revealed insights into factors like RMSD, radius of gyration, RMSF. These comprehensive analyses revealed that ZINC000828320609 possesses superior inhibitory properties.

This breakthrough finding suggests that ZINC000828320609 holds immense promise as a potential drug candidate for treating autism. Its potential to effectively target Cav1.2 makes it a compelling contender in the drug development arena. This research not only offers valuable insights into the inhibitory mechanisms of ZINC000828320609 but also lays the foundation for developing novel therapeutics for autism and potentially other diseases.

The study employs computational methods to virtually screen a large database of compounds for their potential to modulate Cav1.2 channel function based on a AlphaFold-predicted structure. It provides a foundation for further experimental validation and potential drug development for ASD, offering a novel and efficient strategy to target Cav1.2 channels in the treatment of this complex disorder.

For CNS drugs, it is particularly important to evaluate toxicity parameters that can impact brain function and safety. Here, we provide additional toxicity predictions using ADMETLab:

1. hERG Inhibition

- The human ether-à-go-go-related gene (hERG) encodes a potassium channel that is crucial for cardiac repolarization. Inhibition of hERG can lead to prolonged QT interval and potentially fatal arrhythmias. None of the top 10 compounds exhibited predicted hERG inhibition.

1. AMES Toxicity

- AMES test is used to detect mutagenicity, which can be a concern for long-term drug safety. Some of the compounds showed positive AMES toxicity, indicating potential mutagenicity. However, ZINC000828320609 (Lig9) passed this test.

1. Carcinogenicity

- Carcinogenicity assessment is critical for drugs intended for chronic use. Several compounds showed predicted carcinogenicity, highlighting the importance of further toxicological evaluation. Again, ZINC000828320609 (Lig9) was negative for carcinogenicity.

1. CNS penetration

- CNS penetration is a key pharmacokinetic parameter for CNS drugs. While not directly predicted by ADMETLab, we can infer that compounds with favorable LogPo/w and MW values are more likely to penetrate the blood-brain barrier (BBB).

1. Neurotoxicity

- Neurotoxicity can manifest as adverse effects on brain function, behavior, or development. While direct neurotoxicity prediction is challenging, we can evaluate potential neurotoxic liabilities based on structural alerts and known toxicities of similar compounds. None of the top 10 compounds exhibited obvious structural alerts for neurotoxicity.

To alleviate off-target effects due to the structural similarities, experimental strategies such as **Subtype-Selective Tools,** including employment of subtype-specific inhibitors (e.g., ω-agatoxin for P/Q-type, SNX-482 for R-type) to isolate target channel contributions in electrophysiological recordings and genetic models (e.g., Cav2.2 knockout mice for N-type channel studies), **Binding Specificity Assays,** including in silico docking studies across Cav1.2 vs. Cav3.1 and Surface Plasmon Resonance (SPR) to quantify ligand-channel interactions, and **Functional Readouts** (Concentration-response curves in heterologous expression systems (HEK293T cells)) can be employed in the future work.

## Supporting information

S1 Table Docking result of Cav1.2. (XLSX)

S2 Table ADMET of Cav1.2. (XLSX)

S3 Table Pressure profile during NPT equilibrium. (XLSX)

S4 Table Density profile during NPT equilibrium. (XLSX)

S5 Table Profile of gyration of radius during. MD. (XLSX)
