# Correction: Targeted drug screening for autism based on Cav1.2 calcium ion channel

**DOI:** 10.1371/journal.pone.0345218

**Published:** 2026-03-17

**Authors:** Chao Liu

The following information is missing from the Funding statement: This work is supported by Dongguan City University (KY2024004301 to C. L.).
